# DNA Gene’s Basic Structure as a Nonperturbative Circuit Quantum Electrodynamics: Is RNA Polymerase II the Quantum Bus of Transcription?

**DOI:** 10.3390/cimb46110721

**Published:** 2024-10-30

**Authors:** Raul Riera Aroche, Yveth M. Ortiz García, Esli C. Sánchez Moreno, José S. Enriquez Cervantes, Andrea C. Machado Sulbaran, Annie Riera Leal

**Affiliations:** 1 Department of Research in Physics, Division of Natural Sciences and Mathematics, University of Sonora, Hermosillo 83000, Mexico; rriera@cifus.uson.mx; 2 Research and Higher Education Center of UNEPROP, Hermosillo 83105, Mexico; yveth.ortiz@academicos.udg.mx (Y.M.O.G.); eslicamila@gmail.com (E.C.S.M.); sauloenriquezc@gmail.com (J.S.E.C.); andrea.machado5223@academicos.udg.mx (A.C.M.S.); 3 Institute of Research in Dentistry, Department of Integral Dental Clinics, University Center of Health Sciences, University of Guadalajara, Guadalajara 44100, Mexico; 4 Department of Dermatology, General Hospital of the State of Sonora, Hermosillo 83000, Mexico; 5 Childhood and Adolescence Cancer Research Institute, University Center of Health Sciences, University of Guadalajara, Guadalajara 44100, Mexico

**Keywords:** gene basic structure as a circuit, DNA base pairs as RF SQUIDs, RNA pol II the bus of transcription

## Abstract

Previously, we described that Adenine, Thymine, Cytosine, and Guanine nucleobases were superconductors in a quantum superposition of phases on each side of the central hydrogen bond acting as a Josephson Junction. Genomic DNA has two strands wrapped helically around one another, but during transcription, they are separated by the RNA polymerase II to form a molecular condensate called the transcription bubble. Successive steps involve the bubble translocation along the gene body. This work aims to modulate DNA as a combination of *n*-nonperturbative circuits quantum electrodynamics with nine Radio-Frequency Superconducting Quantum Interference Devices (SQUIDs) inside. A bus can be coupled capacitively to a single-mode microwave resonator. The cavity mode and the bus can mediate long-range, fast interaction between neighboring and distant DNA SQUID qubits. RNA polymerase II produces decoherence during transcription. This enzyme is a multifunctional biomolecular machine working like an artificially engineered device. Phosphorylation catalyzed by protein kinases constitutes the driving force. The coupling between n-phosphorylation pulses and any particular SQUID qubit can be obtained selectively via frequency matching.

## 1. Introduction

Structure–function transcription studies revealed a highly evolutionary conserved process for all three life divisions. Transcription is divided into three steps: promoter DNA binding and RNA initiation; processive RNA chain elongation; and termination [1]. Key points of each step were elucidated, including the nucleotide addition cycle (NAC) and the RNA proofreading mechanism after recognizing errors [2]. Also, a similar architecture, active center, and transcription mechanism of cellular RNA polymerases (RNA pol) have been described [3]. Using nucleoside triphosphate (NTP) substrates, RNA pol synthesizes the RNA complementary to the DNA template strand. The transcription process occurs spatially, forming biomolecular condensates, and RNA pol II can be incorporated into them [2,4].

The qubit, the elementary block for quantum information processing [5], is equivalent to a two-level quantum system (TLS) [6]. Some systems have qubit properties, such as nuclear, atomic, molecular spins, electric dipoles [7], and DNA base pairs (bps), naturally. Previously, we carried out a theoretical approach to DNA as a quantum computer [8]. In DNA qubits, Adenine (A), Thymine (T), Cytosine (C), and Guanine (G) are paired superconductors with the central hydrogen bonds (H-bond), acting as a Josephson Junction (JJ). The quantum tunneling of the H-bond in DNA and RNA has been described [9]. Qubits have to store information, be able to be manipulated, and the data must be transferred effectively (quantum bus). Maintaining the quantum coherence of qubits is a critical challenge. The system must be insensitive to external disturbances [10].

The central idea for multiple qubit architectures is based on coupling them to a transmission line and selecting from the frequency at which qubits couple together [11,12]. These behave as nonlinear resonators in which the fundamental and excited levels can be isolated. The general scheme of a coplanar microwave transmission line consists of a one-dimensional cable evaporated on an insulating substrate with two superconducting planes parallel to it on the same surface [13,14]. A finite transmission line is obtained by interrupting the central wire at two points to obtain a resonator that supports a quantized electromagnetic mode, behaving as a harmonic [15].

Cavity Quantum Electrodynamics (cavity QED) studies an atom as a TLS and the quantized electromagnetic field within an optical cavity [16]. Later, Circuit Quantum Electrodynamics (circuit QED) emerges, in which superconducting qubits and microwave transmission lines called resonators replace the atoms and the cavity, respectively [13,17]. A TLS and a coupled harmonic oscillator exist in the cavity QED and the circuit QED [16,17,18]. Superconducting resonators are crucial elements in a circuit QED [17]. A microwave resonator can be an excellent quantum bus for qubit manipulation and memory [11]. In the cavity QED, the atom couples to the electromagnetic field through its electric dipole moment, while superconducting qubits have several coupling alternatives [19]. Circuit QED is a well-suited platform for quantum data due to its flexibility, scalability, and tunability [20]. Experimental works have demonstrated the strong coupling between superconducting qubits and a microwave resonator [21].

Developing a molecular circuit engineering that controls the haphazard states of individual molecular events is a big challenge. In this work, an electrical representation of DNA is carried out following the Quantum Electrodynamics Theory of Circuits and considering the results previously published by our team [8]. A gene is described as a problem of a qubit capacitively coupled to two transmission guides subject to a harmonic forcing in the magnetic flux that passes through it for a frequency $\omega_{r}$ and amplitudes A. A–T, T–A, C–G, and G–C bps are structurally similar to the Radio Frequency Superconducting Quantum Interference Device (RF SQUID), where each level is coupled to a different transmission line. In a resonant regime, the separation between the two energy levels of the qubit is in resonance with the frequency of the transmission lines. Entangled qubit–photon states are generated, and both systems could exchange excitations. We consider genes to be a combination of *n* single-mode microwave resonators and cavities coupling in parallel. The frequency matching can be selectively obtained by coupling the phosphorylation (P) pulse emitted by RNA pol II and any particular SQUID bps during transcription.

This document represents a first step toward establishing the DNA gene’s basic structure as a nonperturbative circuit QED. The outline of this paper is as follows: in Section 2.1, we reviewed the global DNA and transcription analysis. Section 2.2 presents the H-bonded bases as RF SQUIDs and defines the Hamiltonian scheme. Section 2.3 assesses the DNA backbone as a finite transmission line with distributed parameters and the corresponding Hamiltonian. Section 2.4 performs a global analysis to establish DNA as a circuit QED. A quantum description of transcription is proposed in Section 2.5. In the Section 3, we discussed the theoretical background that underlies our model definition. We concluded that every gene in DNA can be modeled as a combination of n circuits.

## 2. DNA Gene’s Basic Structure as a Nonperturbative Circuit Quantum Electrodynamics: Quantum Description of Transcription

### 2.1. DNA and Transcription: Structural and Biochemical Studies

Genomic DNA is double-stranded. The H-bonded bases are assembled in parallel along the direction of the helical axis, perpendicular to the helix backbone. The x-ray analysis revealed a regular space of 0.34 nm between the bases and 36° of twist angle about the helical axis. Ten pairs of bases define the helix anatomy of a complete turn every 3.4 nm [22,23]. A repeating sugar (deoxyribose)-phosphate units form the strands’ backbone [23]. Each bps triplet constitutes a codon that carries on the genetic code. However, most of the human genome does not code for proteins. These regions may provide RNA molecules and transcription factors’ specific sequences of binding sites and exert regulatory functions [24]. The minor and major grooves are two helical spaces with different widths. The first one is delimited by the space when the two antiparallel DNA strands run closest together, while the major groove is defined when they are furthest apart [22]. The above information is the DNA B schematic representation, the predominant type in most cell DNA stretches [23].

The eukaryotic transcription cycle arises during three phases. The RNA chain initiation undergoes various steps, recruiting multiple cellular factors, the mediator complex, and the RNA pol II to form a pre-initiation complex (PIC) at core promoter sequences. PIC composition is identical at all yeast promoters and is the rate-limiting step for transcription [25]. After, the RNA pol II accesses the DNA template strand to begin the RNA synthesis. A cellular RNA pol II forms a stable elongation complex (EC) with the DNA template and the RNA transcript for the transcription elongation [26].

**Figure 1:**
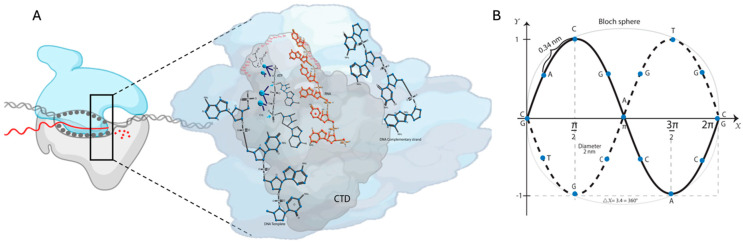
Quantum model of transcription. (**A**) RNA pol II changes the external magnetic flux and generates a phase difference. When the flow phase shift occurs, the Bloch sphere is reproduced. The RNA pol II sends the system pair of pulses (two detuning pulses) with the same parameters (n) times. The driving force is manifested by the processive phosphorylation of alcohol residues in RNA pol II using ATP as the phosphate donor. It produces a rotation of the point on the sphere concerning the rotation axis at the rotation angle. The Bloch sphere can describe Rabi oscillations in a flux qubit. Phosphorylations are applied at the energy-level splitting frequency for the qubit for a specific time with the magnetic-field component along the y-axis. During the pulse, the state vector rotates in the y–z plane about the x-axis. Thus, it extracts the number of times the system was in the state =|0⟩ or |1⟩. (**B**) One-dimensional representation of the DNA strands’ deformation due to the H-bond break to form the Bloch sphere with an applied static magnetic field. A unique point on the sphere’s surface represents any given superposition of the nine states shown. DNA circuit can be expressed as a symmetric and antisymmetric functions: y=Asinx  and y=−Asinx.

Eukaryotic polymerases comprise an evolutionarily conserved catalytically competent ten-subunit core and up to seven peripheral subunits. The C-terminal domain (CTD) of the RNA pol II largest subunit has a repeated heptad motif (Tyr_1_Ser_2_Pro_3_Thr_4_Ser_5_Pro_6_Ser_7_) [27]. The copy number depends on the organism’s complexity (26 in yeast vs. 52 in humans). CTD’s dynamic P and dephosphorylation coordinate the specific association and release of transcriptional factors at every stage of transcription (Figure 1A) [28,29]. For example, transcription initiation and promoter escape at the 5′-end are facilitated by Ser_5_-P for Kin28/Cdk7, while for the elongation, Ser_5_-P is by Srb10/Cdk8 and collaborates Ser_2_-P by Ctk1/Cdk9. The termination and the 3′-end processing are because of the Ser_2_ to Ser_2_-P transition by Bur1/Cdk9 [30,31]. A similar P-process regulates the biological activity of many proteins.

In most promoters, the two primary recognition consensus sequences TTGACA and TATAAT (−35 and −10 hexameric boxes) are separated by a region of 17 ± 1 bps called a spacer [32,33]. The −35 element interacts with the RNA pol II sigma (σ) σ4 domain and the DNA major groove by inserting a helix-turn-helix (HTH). The −43 to −38 sequences interact with the DNA minor grove and the α-CTD [34]. During elongation, the transcription bubble has a 12 ± 1 nucleotide constant size and 8 ± 1 bps RNA–DNA hybrid until RNA pol II reaches a termination codon [35]. RNA pol II selects the correct nucleotide, adds it to the growing RNA chain 3′-end, and releases pyrophosphate (PPi). Identical residues contacting the nucleotide exist in all RNA pols, consistent with a general NAC [36]. The NTP substrate attaches transiently to the open active center enzyme conformation. Structural analysis of the EC revealed a nine-bps DNA–RNA hybrid duplex from the active center cleft at the floor [2,37,38].

In the transcription bubble, DNA is unwound before the RNA pol II catalytic site, allowing the template single strand to reach it and form the DNA–RNA hybrid duplex. It is then rewound to assemble the exiting DNA duplex. Within the unwound region, the growing RNA is attached to the active center cleft with its 3′-end and forms the nine-bps hybrid duplex with the DNA template strand [26]. The high EC stability depends on the DNA–RNA tight binding, which facilitates transcription processivity. Again, CTD-P plays an important role. Spt_5_ spans the RNA pol II cleft from the clamp and contacts the upstream DNA and the non-template DNA strand [39]. Spt_5_ positive charges interact with the negatively charged DNA non-template strand to prevent the bubble’s collapse. The Spt_5_ NGN domain links to all polymerases clamp in the coiled coil [36], indicating an evolutionary conserved binding mode.

### 2.2. A–T, T–A, G–C, and C–G Base Pairs as RF Squids: The Hamiltonian Scheme

The superconducting qubits steam as a leading technology platform for quantum computing [11,40]. Flux qubits exploit magnetic flux through a superconducting loop. A superconducting ring is interrupted by one or more JJs and is crossed by a magnetic flux based on the Josephson effect [11]. The qubit’s quantum state is encoded in the persistent loop current and manipulated by altering the magnetic flux. Suppose that an external oscillating flow of fixed frequency and variable amplitude is applied. In that case, the qubit can be forced by generating oscillations in the populations of its two energy levels through the Landau–Zener–Stuckelberg Transitions.

**Figure 2:**
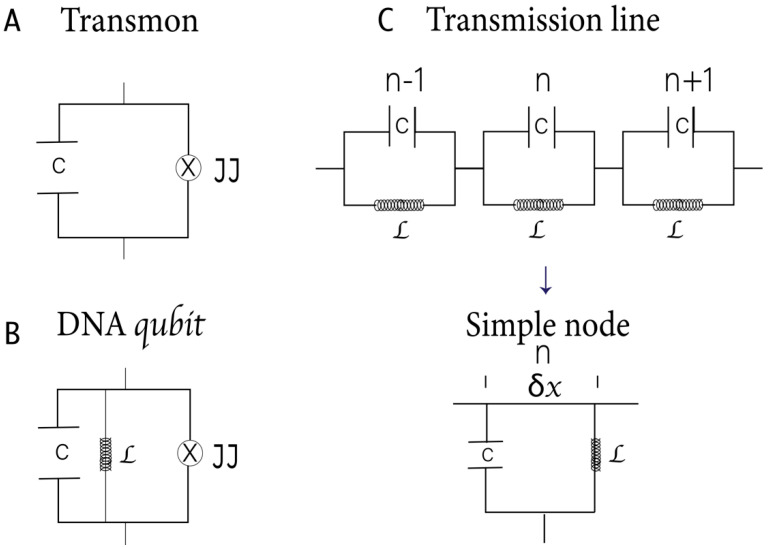
Graphic representation of two qubits and two transmission lines: (**A**) Trasmon; (**B**) DNA qubit. C indicates the capacitor capacity, L, the inductance, and ⊗, the Josephson Junction; (**C**) A typical finite transmission line with n − 1, n, and n + 1 nodes. A single node is represented.

Phase qubits store quantum information in the difference in phases between the JJ on both sides. The quantum states coherent rotations can be induced by applying external microwave pulses [41]. One of the most used systems in circuit QED is the charge qubit (Cooper pair box) coupled through the electrical component of the field to a superconducting coplanar waveguide resonator [40]. Recently, many studies have highlighted the central role of transmon qubits (Figure 2A) in quantum computing [19]. In them, the superconducting island is controlled by an external magnetic field that induces a magnetic flux across the JJs, affecting the superconducting phase difference [11,13].

An anharmonicity with a high energy difference between consecutive levels ensures well-defined qubits and long-lasting quantum calculations. In a previous study, our working group established the role of DNA as a perfect quantum computer by characterizing Adenine (A), Thymine (T), Cytosine (C), and Guanine (G) nitrogenous bps as superconducting islands. In their mutual connection, A–T and C–G represent the entangled quantum states while retaining different structures and morphologies. In addition, we defined DNA qubits by combining the system’s quantum state with classical information [8]:

$$\mathrm{If}\;\left|\left.\psi \right\rangle \right.AT=\left|\left.\psi \right\rangle \right.TA=\left|\left.\psi \right\rangle \right.X,\;\mathrm{then},\;\left|0\right\rangle \left[AT\right]=|0\rangle \left[TA\right]=|0\rangle \left[X\right]$$

$$\mathrm{And}\;\mathrm{if}\;\left|\left.\psi \right\rangle \right.CG=\left|\left.\psi \right\rangle \right.GC=\left|\left.\psi \right\rangle \right.Y,\;\mathrm{then},\;|1\rangle \left[GC\right]=|1\rangle \left[CG\right]=|1\rangle \left[Y\right]$$

A–T and C–G are superconducting qubits with intrinsically quantum behavior. They have quantized energy levels, so their separation is more significant than thermal fluctuations and can be in superposition states. The JJ introduces anharmonicity to the system [11], thus obtaining non-equispaced energy levels. Each transition has a distinct frequency because of the decreasing energy spacing as the quantum number progressively increases. If a magnetic flux is externally applied, the double-well potential and the two eigenfunctions become symmetrical during the antisymmetrical superpositions of the two basis states. In these superconducting rings, a single total wave function describes the state of all electron and hole pairs in the condensate:

$$\psi_{\vec{p}}=\psi e^{i\vec{p}.\vec{r}∕\hbar }.$$

$\vec{p}$ is a single-electron and hole-pair momentum.
${\left|\psi \right|}^{2}$ is the probability density of finding an electron and hole pair in a given volume.
$\vec{p}.\vec{r}∕\hbar$ of the phase associated with each electron and hole pair in space.

Because this wave function must be unvalued, the accumulated phase change (∆∅), when traveling a closed path around the year, must be a multiple of 2π:

∆∅=2nπ

where n is an integer number.

Because the RNA pol II contacts only one element of the pair (classical information) in the template strand, we considered the number of aromatic rings to achieve one of the four Bell states [8]:

$$|0\rangle \left[\frac{A}{T}\right]\;\mathrm{or}\;|0\rangle \left[\frac{T}{A}\right]\;\mathrm{and}\;\left|\left.1\right\rangle \left[\frac{C}{G}\right]\right.\;\mathrm{or}\;\left|\left.1\right\rangle \left[\frac{G}{C}\right]\right.$$

Purines have two rings =|0⟩; Pyrimidines have one ring =|1⟩. Then, RNA pol II interacts with one component of the pair in the template strand.

|ψ⟩A=|ψ⟩G=|0⟩, and |ψ⟩T=|ψ⟩C=|1⟩, then

$$|0\rangle \left[\frac{A}{T}\right]=|0\rangle \left[01\right],\;|0\rangle \left[\frac{T}{A}\right]=|0\rangle \left[10\right],\;\mathrm{and}\;\left|1\right\rangle \left[\frac{G}{C}\right]=\left|1\right\rangle \left[01\right],\;\left|1\right\rangle \left[\frac{C}{G}\right]\left|1\right\rangle \left[10\right]$$

$$|\psi \rangle AT=\left[00\right],\;|\psi \rangle TA=\left[01\right],\;|\psi \rangle CG=\left[11\right],\;\mathrm{and}\;|\psi \rangle GC=\left[10\right]$$

A JJ forms the foundation of a SQUID. However, these junctions also present an internal capacitive effect because they are constituted by two superconducting electrodes separated by an insulator [11]. The JJ can be considered a linear harmonic LC circuit with a nonlinear inductance. Two types of SQUIDs are commonly used in quantum computer applications: a DC SQUID, which contains two parallel junctions, and an RF SQUID, which includes only one junction. In DNA qubits, the central H-bond, acting like a JJ [8], forms the foundation of the RF SQUIDs (Figure 2B). The H-bond can induce strong inter- or intra-molecular electronic coupling by enhancing resonance, electron delocalization, or planarizing the conjugated backbone.

Nitrogenous (N) number three (N3) of a T in T–A bps has a lone pair of electrons as part of the π-cloud. The N1 in A uses the lone pair to attract the H attached to the N3 of T. Similarly, in C–G bps, the N3 in C uses its lone pair of electrons to form an H-bond with the N1 of G. In our previous work, we explained, using physics approximations, how electrons interchanged a biological quantum of energy to form electrons and hole pairs in the JJ. The electron (e) three ($e_{3}$) with the momentum P+K emits a biology boson ($B_{b}=\hbar \omega$) to $e_{2}$ losing the momentum K. Then, it occupies the position of the hole (h) one ($h_{1}$) with momentum P. Then, $e_{1},$ through the Tunnel Effect, passes through the barrier (NH–N) to the position of $e_{3}$, where there is now a hole (Figure 4 in the reference [8]).

The first Josephson e bold quation ($I_{s}=I_{0}sin\left(\emptyset_{2}-\emptyset_{1}\right))$ describes how the tunneling current depends on the two superconductors’ phase difference. The second Josephson equation ($\frac{d}{dt}\left(\emptyset_{2}-\emptyset_{1}\right)=\frac{qV}{\hbar })$ represents the time evolution of the phase difference given an external voltage [11]. V is the potential difference in the JJ. These two equations describe the temporal phase evolution of the two superconductors. If a constant voltage is applied to a JJ, the phase difference will evolve linearly in time (AC current). As opposed, if we use no external voltage, the phase difference becomes constant (constant supercurrent despite no external voltage) due to the phase coherence of the pairs. It is known as the DC Josephson effect.

$$\mathrm{If}\;\emptyset =\emptyset_{2}-\emptyset_{1},\;\text{then}$$

$$I_{s}=I_{0}sin\left(\emptyset \right)\;\mathrm{and}\;V=\frac{h}{4\pi e}\frac{d\emptyset }{dt}$$

$$P=\frac{dE_{jj}}{dt}\;\mathrm{with}\;E_{jj}=\int Pdt=\int VI_{s}dt=\int \frac{h}{4\pi e}\frac{d\emptyset }{dt}I_{0}sin\left(\emptyset \right)dt$$

$$\mathrm{Then},\;E_{jj}=\frac{h}{4\pi e}I_{0}cos\emptyset ,\;\mathrm{where}\;E_{j}=\frac{h}{4\pi e}I_{0}$$

$$\mathrm{Then},\;cos\emptyset =1-\frac{1}{2}\emptyset^{2}-\frac{1}{4!}\emptyset^{4}+O(\emptyset^{6})$$

The first term $E_{j}$ can be disregarded because it is a constant factor, while the terms of order $O(\emptyset^{6})$ can be disregarded because ∅ is small.

The Hamiltonian is an energy operator containing kinetic energy $K=\frac{P^{2}}{2m}$ and potential energy of the form $V\left(r\right)$.

$$\mathrm{Then},\;H=\frac{P^{2}}{2m}+V\left(r\right)$$

The Hamiltonian of a qubit with a single JJ is given by

$$H=\frac{Q^{2}}{2C}+U\left(\Phi \right)\;\mathrm{with}\;U\left(\Phi \right)=\frac{\Phi^{2}}{2L}-E_{j}cos\left[\frac{2\pi }{\Phi_{0}}\left(\Phi -\Phi_{ext}\right)\right]$$

$$\emptyset =2\pi \frac{\Phi }{\Phi_{0}}$$

$$n=\frac{Q}{2e}\;\mathrm{and}\;E_{C}=\frac{e^{2}}{2C}$$

$$H=\frac{Q^{2}}{2C}+\frac{\Phi^{2}}{2L}-E_{j}cos\left[\frac{2\pi }{\Phi_{0}}\left(\Phi -\Phi_{ext}\right)\right]$$

(1) $$H=4E_{C}n^{2}+E_{j}\left(\frac{1}{2L}+\frac{4\pi }{{\Phi_{0}}^{2}}\right)\Phi^{2}+E_{j}\frac{2}{3{\Phi_{0}}^{4}}\Phi^{4}$$

Φ: flow in the JJ;
$\Phi_{ext}$: external magnetic flux; $\Phi_{ext}=0$;
C: capacitance;
L: inductance;
$E_{j}$: Josephson energy;
$\Phi_{0}$: magnetic flux quantum;
$E_{jj}$: JJ energy.

Equation (1) is the Hamilton equation. Following the first quantization procedure, the two conjugate variables n and Φ are promoted to non-commuting operators.

$$n=\hat{n}\;y\;\Phi =\hat{\Phi }$$

(2) $$\hat{H}=4E_{C}{\hat{n}}^{2}+E_{j}\left(\frac{1}{2L}+\frac{4\pi }{{\Phi_{0}}^{2}}\right){\hat{\Phi }}^{2}+E_{j}\frac{2}{3{\Phi_{0}}^{4}}{\hat{\Phi }}^{4}$$

The first two terms in Equation (2) represent harmonic oscillators, and the third term is the anharmonicity.

Introducing the standard annihilation ${\hat{a}}^{-}$ and creation ${\hat{a}}^{+}$ operators are helpful.

$${\hat{a}}^{+}=\frac{1}{\sqrt{2}}\left(\frac{1}{\sqrt{\beta }}\hat{\Phi }+i\sqrt{\beta }\hat{n}\right)$$

$${\hat{a}}^{-}=\frac{1}{\sqrt{2}}\left(\frac{1}{\sqrt{\beta }}\hat{\Phi }-i\sqrt{\beta }\hat{n}\right)$$

$$\mathrm{With}\;\beta =\sqrt{8\frac{E_{C}}{E_{j^{*}}}}\;\mathrm{where}\;E_{j^{*}}=\frac{1}{2}\left(\frac{1}{L}+\frac{8\pi }{{\Phi_{0}}^{2}}\right)E_{j}$$

The action of ${\hat{a}}^{+}$ is to create a quantized excitation of the flux and charge degrees of freedom of the magnetic and electric fields. It creates a photon of frequency $\omega_{r}$. It is stored in the circuit.

In function of ${\hat{a}}^{+}$ and ${\hat{a}}^{-}$:

$$\hat{\Phi }=\sqrt{\frac{\beta }{2}}\left({\hat{a}}^{-}+{\hat{a}}^{+}\right)\;\mathrm{and}\;\hat{n}=\frac{i}{\sqrt{2\beta }}\left({\hat{a}}^{-}-{\hat{a}}^{+}\right)$$

Then, the Hamiltonian in Equation (2) combines in

(3) $$\hat{H}=\omega_{r}\left({\hat{a}}^{+}{\hat{a}}^{-}+\frac{1}{2}\right)+\frac{\alpha }{12}{\left({\hat{a}}^{+}+{\hat{a}}^{-}\right)}^{4}$$

With $\omega_{r}=\sqrt{8E_{C}E_{j^{*}}}$ the new frequency and the anharmonicity $\alpha =-E_{C}$.

Note that the Hamiltonian represented in Equations (2) and (3) corresponds to our qubit proposal based on the DNA characteristics and differs from those described in other works (Figure 2B).

### 2.3. DNA Backbone as a Finite Transmission Line with Distributed Parameters: The Hamiltonian Scheme

A transmission line is a structure of uniform geometry used to transport electrical signals or RF energy efficiently from one point to another [11]. It is also a medium that propagates information through electromagnetic waves at very high frequencies [42]. Because it behaves like a cable that spreads energy, it can be analyzed like a quantum oscillator electrical circuit based on its frequency response. The circuit is considered a line with distributed parameters at high frequencies since it has dimensions comparable to the wavelength [43]. Thus, the circulated current’s amplitude and phase differ at every point. The wavelength is very short for high frequencies, so the transmission lines behave like resonant circuits for a specific frequency range.

Two parallel conductors form pair lines. Its variants are used in telephony and data transmission [44,45]. Electromagnetic energy propagates around the conductors of the line. If a voltage Vs is applied to a pair line, an electric (E) field is generated between the conductors as opposite charges accumulate. The Vs makes an electric current $\left(Ι\right)$ flow through the line’s conductors terminated at a load impedance ($Z_{L}$). This current also generates a magnetic H field around the conductors. The direction of the current and fields is reversed in each half-cycle of the voltage [45]. By multiplying H by μ, we obtain the induction magnetic vector (B), and then, by integrating B over a plane parallel to the wires, we obtain the magnetic flux (Φ) that “joins” the circuit.

The mathematical definition of a circuit inductance is $L=\frac{\Phi }{Ι}$. If we have a transmission line length $x_{0}$ units long, and if that line has a distributed inductance L, then the inductance is $L=Lx_{0}$. Two conductors with charge ±Q separated by a distance generate a potential difference (V). $Q=\pm \left(\rho x_{0}\right)$ in every section of a transmission line length $x_{0}$. Where ρ is the lineal distribute charge. The primary transmission line parameters are its capacitance (C) and inductance (L) per unit length, the impedance ($Z_{0}$), and the mode frequency (ω) [21]. These parameters are related by

$$Z_{0}=\sqrt{\frac{L}{C}}\;\mathrm{and}\;\omega =\frac{1}{\sqrt{LC}}$$

The electric and magnetic fields are transverse to the direction of propagation. It is called Transverse Electromagnetic Waves (TEM) [46]. We can distinguish between infinite, semi-finite, and finite transmission lines. The latter is obtained by interrupting the central cable at two points so that a resonator that supports a quantized electromagnetic mode behaving like a harmonic oscillator is obtained. A resonator is a structure that encloses oscillatory electromagnetic energy without spreading it [11]. Boundary conditions restrict the wave configuration inside to patterns with integral numbers of half or quarter wavelengths along a propagation axis [45]. Therefore, specific discrete resonant frequencies $\omega_{n}$ are allowed. There is no energy dissipation if the resistive element is in series at zero current or parallel at zero voltage. Typical TEM resonators contain lossless elements at their end. Resonators are widely used to manipulate signals and energies, acting as bandpass filters, allowing only for the frequencies close to the desired resonant frequency ($\omega_{n}$) or as band-stop filters, letting all frequencies pass [21].

Characterizing single-stranded DNA as a transmission line is also of great interest. Single-stranded DNA can exhibit rich electrical properties. In the DNA backbone (formed by phosphate groups and deoxyriboses), C–N bonds attach deoxyribose to the bps [47]. The negative charges at the phosphate groups connect bps and give structural support for the double helix. Small polaron-assisted hopping between the next nearest neighbors of the DNA molecular wire was suggested as the transport mechanism responsible for the solid high-temperature dependence of the electrical conductivity [48]. Our DNA wire model has connections between successive bps, which are the only sites included. Porath and Cols demonstrated that neither sugar–phosphate backbones nor the specific bps sequence mediated the long-distance conduction in DNA [49]. Besides the multiple efforts to study the electrical current through single DNA fragments, the conduction mechanism, especially in long DNA molecules, still needs to be established.

Figure 2C shows a schematic representation of DNA based on the model of parameters distributed in a transmission line. The line length is proportional to the total capacitance and inductance. In this model, the backbone phosphate groups carry the inductive effect, while the bases have the capacitive effect. Since it is a superconducting circuit, we will ignore the electrical resistance and magnetic fields within the strands, which are expelled due to the Meissner effect [8,50].

Using the Telegrapher equations, we can obtain the transmission line equation and assume that the DNA strand acts as a lossless line with R=0 and G=0, where R and G are the resistance and shunt admittance per unit length [51]. The first equation

$$\frac{\partial V\left(x,t\right)}{\partial x}=-\left(L\frac{\partial Ι\left(x,t\right)}{\partial t}\right)$$

explains the voltage dependence of the distributed inductance L multiplied by the time derived from the current flowing in the line at one point. The second equation

$$\frac{\partial Ι\left(x,t\right)}{\partial x}=-\left(C\frac{\partial V\left(x,t\right)}{\partial t}\right)$$

explains that the current loss as we go down the line is proportional to the distributed capacitance C multiplied by the time rate of the voltage change in the line.

An LC oscillator is characterized by its inductance L, capacitance C, angular frequency $\omega_{r}=\frac{1}{\sqrt{LC}}$, and impedance $Z_{r}=\sqrt{\frac{L}{C}}$. The oscillator energy is given by

(4) $$H_{LC}=\frac{Q^{2}}{2C}+\frac{\Phi^{2}}{2L}$$

As $I=\frac{dQ}{dt}$, and $Q\left(t\right)=\int_{0}^{t}I\left(t^{⋆}\right)d\left(t^{⋆}\right)$, then

(5) $$H_{LC}=\frac{Q^{2}}{2C}+\frac{1}{2}C{W_{r}}^{2}\Phi^{2}$$

where, by Faraday’s law

$$\Phi \left(t\right)=\int_{0}^{t}V\left(t\right)d\left(t\right)$$

Equation (5) is an LC harmonic oscillator equivalent to the coordinate mechanical harmonic oscillator Φ

P=Q and M=C

$$\mathrm{Then},\;\left[\Phi ,Q\right]=i\hbar$$

Introducing the creation and annihilation operators (${\hat{a}}^{+}$ and ${\hat{a}}^{-}$)

$$\hat{\Phi }=\Phi_{zpf}\left({\hat{a}}^{+}+{\hat{a}}^{-}\right)\;\;\mathrm{and}\;\hat{Q}={iQ}_{zpf}\left({\hat{a}}^{+}-{\hat{a}}^{-}\right)\;\text{with}$$

$$\Phi_{zpf}=\sqrt{\frac{\hbar }{2W_{r}C}}=\sqrt{\frac{\hbar Z_{r}}{2}}\;\mathrm{and}\;Q_{zpf}=\sqrt{\frac{\hbar W_{r}C}{2}}=\sqrt{\frac{\hbar }{2Z_{r}}}.\;\text{Then},$$

$${\hat{H}}_{LC}=\hbar W_{r}\left({\hat{a}}^{+}{\hat{a}}^{-}+\frac{1}{2}\right)$$

where ${\hat{a}}^{+}{\hat{a}}^{-}|n\rangle =n|n\rangle$ with n=0,1,2,… and $\frac{1}{2}$ is the zero-order energy or null energy.

${\hat{a}}^{+}=\sqrt{\frac{\hbar Z_{r}}{2}}\left(\hat{\Phi }-iZ_{r}\hat{Q}\right)$ creates a quantum excitation of flow and charge equivalent to the electric and magnetic fields (a frequency photon $\omega_{r}$ inside the circuit).

We can generalize the result for a transmission line in the x direction and length d. The energy associated with each capacitor is $\frac{{Q_{n}}^{2}}{2C_{0}}$, and to each inductor, $\frac{{\left(\Phi_{n+1}-\Phi_{n}\right)}^{2}}{2L_{0}}$. Where $\Phi_{n}$ is the variable flow associated with node n, and $Q_{n}$ the conjugate variable is the charge at node n.

Then, the Hamiltonian for the discrete linear transmission resonator associated with Figure 2C is

$$H=\sum_{n=0}^{N-1}\left[\frac{1}{2C_{0}}{Q_{n}}^{2}+\frac{1}{2L_{0}}{\left(\Phi_{n+1}-\Phi_{n}\right)}^{2}\right]$$

$L_{0}\;$ and $C_{0}$ are the variable inductance and capacitance associated with each node n and flow $\Phi_{n}$.

In the continuous limit, considering $\delta_{x}$ is the size of a unit cell that tends to zero,

$C_{0}=\delta_{x}c_{0}$ and $L_{0}=\delta_{x}l_{0}$ with $c_{0}$ y $l_{0}$ the linear density of capacitance and inductance.

Defining a continuous flow field as $\Phi \left(x_{n}\right)=\Phi_{n}$, the charge as $Q\left(x_{n}\right)=\frac{Q_{n}}{\delta_{x}}$, and taking the $\delta_{x}$ and d=N∆X constant, the Hamiltonian for a continuous linear transmission resonator is

(6) $$H=\int_{0}^{d}dx\left[\frac{1}{2c_{0}}Q^{2}\left(x\right)+\frac{1}{2l_{0}}{\left(\partial_{x}\Phi_{x}\right)}^{2}\right]$$

where $\partial_{x}\Phi_{x}=\frac{\left(\Phi_{n+1}-\Phi_{n}\right)}{\delta_{x}}$.

The charge $Q\left(x,t\right)=c_{0}\partial_{t}\Phi \left(x,t\right)$ is the generalized canonical moment of the flow $\Phi \left(x,t\right)=\int_{0}^{t}V\left(x,t\right)dt$ with $V\left(x,t\right)$, the voltage.

Using the information above, we obtain the wave propagation equation along the linear transmission resonator:

(7) $${v_{0}}^{2}\frac{\partial^{2}\Phi \left(x,t\right)}{\partial x^{2}}-\frac{\partial^{2}\Phi \left(x,t\right)}{\partial t^{2}}=0$$

${v_{0}}^{2}=\frac{1}{l_{0}c_{0}}$ is the speed of light in the biological medium of DNA.

The Equation (7) solution is expressed as a linear combination of normal nodes of a size $\frac{\lambda }{2}$.

$$\Phi \left(x,t\right)=\sum_{m=0}^{\infty }U_{m}\left(x\right)\Phi_{m}\left(t\right)$$

with ${\ddot{\Phi }}_{m}=-{\omega_{m}}^{2}\Phi_{m}$ a differential equation of an oscillating function in time at the frequency node $\omega_{m}$

$$U_{m}\left(x\right)=A_{m}cos\left(K_{m}x+\varphi_{m}\right)$$

with amplitude $A_{m}$, wave vector $K_{m}=\frac{\omega_{m}}{v_{0}}$, and phase $\varphi_{m}$.

The Hamiltonian given by Equation (6) can be expressed as a discrete Hamiltonian:

(8) $$H=\sum_{m=0}^{\infty }\left[\frac{{Q_{m}}^{2}}{2C_{r}}+\frac{1}{2}C_{r}{\omega_{m}}^{2}{\Phi_{m}}^{2}\right]$$

This Hamiltonian (Equation (8)) represents the sum of simple harmonic oscillators, with $C_{r}=dc_{0}$ as the total capacitance of the resonator and $Q_{m}=C_{r}{\dot{\Phi }}_{m}$ the conjugate charge $\Phi_{m}$.

Following the quantization procedure with the variables $\Phi_{m}$ and $Q_{m}$, we define the creation and annihilation operators:

$${\hat{\Phi }}_{m}=\sqrt{\frac{\hbar Z_{m}}{2}}\left({{\hat{a}}_{m}}^{+}+{{\hat{a}}_{m}}^{-}\right)$$

$${\hat{Q}}_{m}=i\sqrt{\frac{\hbar }{2Z_{m}}}\left({{\hat{a}}_{m}}^{+}-{{\hat{a}}_{m}}^{-}\right)$$

where $Z_{m}=\sqrt{\frac{L_{m}}{C_{r}}}$ is the characteristic impedance of the m node and $\frac{1}{L_{m}}=C_{r}{\omega_{m}}^{2}$.

Then, the Hamiltonian given in Equation (8) finally transforms into

$$\hat{H}=\sum_{m=0}^{\infty }\hbar \omega_{m}{{\hat{a}}_{m}}^{+}{{\hat{a}}_{m}}^{-}$$

where $\omega_{m}=\left(m+1\right)\omega_{0}$, and $\frac{\omega_{0}}{2\pi }=\frac{v_{0}}{2d}$ is the frequency.

### 2.4. DNA as a Quantum Electrodynamic Circuit: The Hamiltonian Scheme

A coplanar waveguide’s (CPW) geometry allows for increased electromagnetic energy density and coupling [20]. The strong coupling permits operations to be carried out on the qubit and even entanglement of multiple qubits in the same cavity. Using two-dimensional CPWs (TD–CPWs) and circuit QED, researchers have realized high-efficiency entanglement of up to 20 qubits and high-fidelity two-qubit gates for achieving up to 64 qubits. The electromagnetic field inside conventional superconducting resonators in circuit QED (including TD–CPWs and 3D-cavities) are standing waves determined by boundary conditions, resulting in a topographic dependence of the fields’ amplitude [21]. In circuit QED, resonant microwave cavities are replaced by sections of microwave transmission lines (integrated circuits) [19]. If superconducting materials are used, the system’s losses and dephasing would be limited only by the system properties [11]. Another way to mitigate losses is to lower the electric field energy stored at interfaces and surfaces to the energy stored in a vacuum. That is why using 3D-microwave cavities or 3D-resonators rather than planar circuits is preferred [17,52]. This is a new quantum information processing prototype with information stored in a cavity.

Here, the effective Hamiltonian for our DNA archetype can be represented as

$$H=H_{RF-S}+H_{R1}+H_{R2}+H_{I_{RFS-R_{1}-R_{2}}}+H_{\kappa }+H_{\gamma }$$

where $H_{RF-S}$ is the Hamiltonian of a flux qubit with a single JJ (A–T, T–A, G–C, or C–G). $H_{R1}$ and $H_{R2}$ represented the Hamiltonian of the two single-stranded DNA separately. To complete the effective representation of the Hamiltonian in DNA, $H_{I_{RFS-R_{1}-R_{2}}}$ represents the interaction between the qubit and two-stranded DNA.

$H_{\kappa }$, the cavity losses and the decay rate.
$H_{\gamma }$, decoherence or decay of the two-level system.

As A–T and C–G are superconductors; their properties limit the system’s losses and dephasing ($H_{\kappa }=H_{\gamma }=0)$.

**Figure 3:**
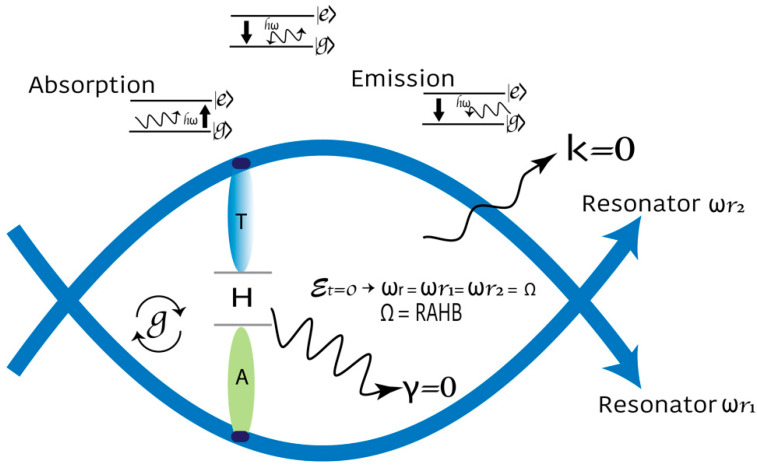
The model of DNA as a 3D-nonperturbative cavity quantum electrodynamics defined by two resonators. In the energy level spectrum of the cavity–qubit system in the zero-detuning case ($\omega_{r}=\Omega )$, a qubit is strongly coupled to a cavity, producing a unique signal when tuned into resonance. We hypothesize that the H-bond provides maximum coupling. RAHB: the resonance-assisted hydrogen bond. The Jaynes–Cummings Hamiltonian assumes the rotating wave approximation ($g\ll \omega_{r},\;\Omega$, $\omega_{r}\sim \Omega )$. The speed of quantum operations is represented by g. To maximize and maintain the coupling g∕π larger than the two main dephasing rates of the system (g≫k,γ) is the target.

According to the Hamiltonian, a strong coupling qubit cavity produces an exceptional footprint when entering resonance. The coherent oscillation between two quantum states is known as Rabi oscillation, and the frequency is the Rabi frequency ($\Omega_{R})$ (Figure 3). The $\Omega_{R}$ is proportional to the energy of the TLS-electromagnetic field interaction [53]. For a flux qubit coupled to a single mode of the electromagnetic field of a transmission line that acts as a resonator, the system’s Hamiltonian consists of three parts: the qubit term ($H_{1}$); the electromagnetic field term confined in the resonator ($H_{2}$); and the Rabi-type interaction term ($H_{3}$).

(9) $$\hat{H}={\hat{H}}_{1}+{\hat{H}}_{2}+{\hat{H}}_{3}=\frac{1}{2}\left(\epsilon \left(t\right)\sigma_{z}+\Delta \sigma_{x}\right)+\hbar \omega_{r}\left({\hat{b}}^{+}{\hat{b}}^{-}+\frac{1}{2}\right)+\hbar g\sigma_{y}\left({\hat{b}}^{+}+{\hat{b}}^{-}\right)$$

$\omega_{r}$ is a resonator resonance frequency.

$\epsilon \left(t\right)=\epsilon_{0}+Acos\omega t$, but in DNA under the ground conditions, Acosωt=0. At this point, we consider the oscillator at a fixed instant, where $\epsilon_{0}$ corresponds to the minimum energy or the fundamental state ($\epsilon_{0}=\frac{1}{2}\hbar \omega$).

Δ corresponds to the two-level difference in energy for $\epsilon \left(t\right)=\epsilon_{0}$,$\;\Delta =\hbar \Omega_{R}$

$$\hat{H}=\frac{\hbar \Omega_{R}}{2}\sigma_{x}+\hbar \omega_{r}\left({\hat{b}}^{+}{\hat{b}}^{-}+\frac{1}{2}\right)+\hbar g\sigma_{y}\left({\hat{b}}^{+}+{\hat{b}}^{-}\right)$$

In the absence of damping, the Hamiltonian can be readily diagonalized. The detuning parameter is $\epsilon \left(t\right)=\Omega_{R}-\omega_{r}$. In the zero-detuning case, $\Omega_{R}=\omega_{r}$.

$\sigma_{x}$, $\sigma_{y}$, and $\sigma_{z}$ are the Pauli matrices.

${\hat{b}}^{+}$ and ${\hat{b}}^{-}$ are photon creation and annihilation operators.

g is the intensity of the qubit–electromagnetic field coupling rate. It depends on the strength of the electric or magnetic field at the system site, its dipole moment, and the system’s properties [52].

(10) $$g=\frac{dA_{e}}{\sqrt{2}\hbar }\;\mathrm{or}\;g=\frac{\mu A_{m}}{\sqrt{2}\hbar }$$

where $A_{e}$ and $A_{m}$ are the electric and magnetic field amplitudes.

Expanding the interaction term in terms of the rise and fall operators, $\sigma_{y}=\sigma_{+}+\sigma_{-}$

$$\hbar g\left(\sigma_{+}{\hat{b}}^{+}+\sigma_{+}{\hat{b}}^{-}+\sigma_{-}{\hat{b}}^{+}+\sigma_{-}+{\hat{b}}^{-}\right)$$

A TLS and a harmonic oscillator are coupled in a circuit QED [21]. The circuit can operate in two limits: on-resonant and off-resonant dispersive regimes. On resonance, the separation between the two energy levels of the qubit resonates with the transmission line frequency. Entangled qubit–photon states are generated, and both system parts can exchange excitations without energy loss [11]. In an ultra-strong coupling regime, the coupling intensity reaches values comparable to the resonator frequency, rendering the Jaynes–Cummings Model invalid. In the dispersive regime, the transmission line frequency differs from the qubit energy levels separation. In this situation, the energy levels of the qubit will depend on the photons and vice versa. Consequently, the resonator could obtain information about the qubit’s state and couple multiple qubits together [54].

Molecular circuits have been developed to control complex functions in biological and biochemical systems. In the DNA ground state, each nitrogenous base is coupled to a transmission line in resonance with the H-bond that joins it to its complementary base connected to a second transmission line. When RNA pol II emits pulses at the end of the transmission line, the system enters a dispersive regime. It is a sub-regime of the Jaynes–Cummings Model that allows for the measurements of the qubit’s state act on the resonator. The energy levels of the qubit are normalized depending on the state of the resonator and vice versa [54]. The qubit state is determined by varying the waveguide frequency until it matches the new frequency of the resonator. It also allows for the manipulation of the qubit by forcing the resonator with a signal sent through the waveguide similar to the natural qubit frequency.

This work approaches the DNA gene structure as the lineal combination of (n) circuit QED. Each level of a bp–flux qubit is coupled to an independent resonator. The frequency of the qubit is similar to that of the resonators. For a flux qubit coupled to two modes of the electromagnetic field of a transmission line that acts as a resonator, the system’s Hamiltonian consists of three parts: the qubit term ($H_{1}$); the electromagnetic field term confined to the two resonators, and the Rabi-type interaction term ($H_{2}$) (modified from Equation (9)):

$$\hat{H}={\hat{H}}_{1}+{\hat{H}}_{2}=\frac{1}{2}\left(\epsilon \left(t\right)\sigma_{z}+\Delta \sigma_{x}\right)+\sum_{i=1}^{2}\left(\hbar \omega_{r_{i}}\left({{\hat{b}}_{i}}^{+}{{\hat{b}}_{i}}^{-}+\frac{1}{2}\right)+\hbar g_{i}\sigma_{y}\left({{\hat{b}}_{i}}^{+}+{{\hat{b}}_{i}}^{-}\right)\right)$$

In the DNA ground state, $\epsilon \left(t\right)=\Omega_{R}-\omega_{r}=o$

$$\hat{H}=\frac{\hbar \Omega_{R}}{2}\sigma_{x}+\sum_{i=1}^{2}\left(\hbar \omega_{r_{i}}\left({{\hat{b}}_{i}}^{+}{{\hat{b}}_{i}}^{-}+\frac{1}{2}\right)+\hbar g_{i}\sigma_{y}\left({{\hat{b}}_{i}}^{+}+{{\hat{b}}_{i}}^{-}\right)\right)$$

**Figure 4:**
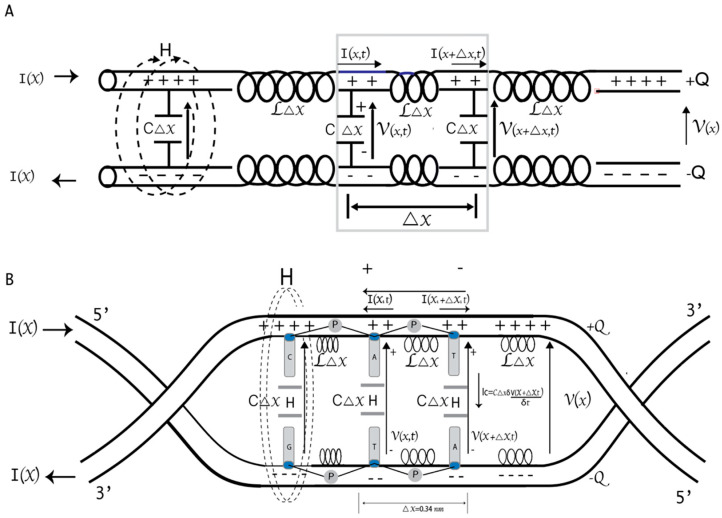
Model of DNA strands as a distributed parameter transmission line. (**A**) Representation of the distributed inductance and capacitance in a transmission line. The line is divided into ∆x sections, each with an inductance L∆x and capacitance C∆x. (**B**) Representation of the distributed inductance and capacitance in DNA strands as transmission lines. There are connections between successive bp. Hence, the distributed sites are the nucleobases. Each level of the DNA qubit is capacitively coupled to a transmission guide, subject to harmonic forcing in the magnetic flux passing through it. The application of Kirchhoff’s laws is represented.

In DNA, A–T, T–A, C–G, and G–C qubits are coupled to a virtual cavity defined by two resonators. This constitutes the DNA basic unit. The difference between the system’s energy levels in the DNA ground state is $\hbar \Omega_{R}$. It is due to the movement of electron and hole pairs in the aromatic ring without an external force [8]. A–T and C–G are strongly coupled to the resonators, which allow for the operations to be performed during the lifetime of the quantum state. The coherent oscillation between the two bps quantum states is equivalent to the phenomenon known as Rabi oscillations. The oscillation frequency is the Rabi frequency. It is related to the energy of the qubit–electromagnetic field interaction. A perturbation can tune the bps levels such that a certain level splitting coincides with the cavity photon energy Figure 4.

Analyzing Equation (10), the average field strength is estimated using the integral of the field amplitude over the space volume delimited by the two resonators. DNA spiral diameter is around 2 nm. Thus, we assume that the space dimensions give us $V=\frac{4}{3}\pi r^{3}$, where V is the sphere’s volume, and r is the radius. Taking a space average and setting that $\epsilon =\epsilon_{0}$, and $\mu =\mu_{0}$ [52],

$$\frac{1}{2}\int \epsilon {\left|A_{e}\right|}^{2}dV=\frac{\hbar \omega_{r}}{2}\;\mathrm{or}\;\frac{1}{2}\int {\frac{\left|A_{m}\right|}{\mu }}^{2}dV=\frac{\hbar \omega_{r}}{2}$$

$$\frac{1}{2}\epsilon_{0}A_{e}^{2}V=\frac{\hbar \omega_{r}}{2}\;\mathrm{or}\;\frac{1}{2\mu_{0}}A_{m}^{2}V=\frac{\hbar \omega_{r}}{2}$$

$$\frac{2}{3}\epsilon_{0}A_{e}^{2}\pi r^{3}=\frac{\hbar \omega_{r}}{2}\;\mathrm{or}\;\frac{2}{3\mu_{0}}A_{m}^{2}\pi r^{3}=\frac{\hbar \omega_{r}}{2}$$

$$A_{e}^{2}=\frac{3\hbar \omega_{r}}{4\pi \epsilon_{0}r^{3}}\;\mathrm{or}\;A_{m}^{2}=\frac{3\mu_{0}\hbar \omega_{r}}{4\pi r^{3}}$$

Then, $A_{e}=\frac{1}{2r}\sqrt{\frac{3\hbar \omega_{r}}{\pi \epsilon_{0}r}}$ for the electric field, and $A_{m}=\frac{1}{2r}\sqrt{\frac{3\mu_{0}\hbar \omega_{r}}{\pi r}}$ for the magnetic field.

In the following section, we will describe the theoretical basis for DNA transcription and compare this process to the dispersive detuning regime.

### 2.5. Quantum Description of Transcription

Despite the progress in elucidating the critical events during transcription, what needs to be addressed is that the process takes place strictly in a spatially defined manner. How the transcription spatial organization is achieved is still an unsolved question. A physiochemical phenomenon called biomolecular condensates is partly formed through phase separation to achieve spatiotemporal regulation of some biological processes [55,56,57,58]. Phase separation is crucial in regulating the transcriptional events at every step of a transcription cycle (Figure 1A) [59,60].

Multivalent interactions constitute driving forces underlying phase separation in biomolecular condensates. They include disordered regions and structured, repeated modular oligomerization domains [61]. Some molecules function as scaffolds to assemble phase-separated structures, whereas client components are recruited by directly binding to scaffolds [55]. In general, modular interaction domains in evolutionarily conserved protein segments create multivalency that facilitates the engagement of multiple binding complexes. The dynamic interacting networks initiate and promote phase separation [60]. In many proteins, the succession of structural components such as tyrosine, glycine, and serine have provided multivalency to undergo phase separation and enable the formation of biomolecular condensates [29]. Unlike the classical transcription describing the RNA pol II tracks along the relatively static DNA template strand to synthesize the nascent RNA, the phase separation model allows for the description of a quantum model of transcription. RNA pol II can be incorporated into transcriptional condensates [58,61]. Transcription bubble translocation along the DNA requires rotation of the RNA pol II around the DNA axis. The linking number density has been calculated using the following equation:

$$\omega_{0}x=\theta +\emptyset \;\mathrm{with}\;\omega_{0}=1.85\;{\mathrm{nm}}^{-1}$$

where $\omega_{0}x$ is the accumulative rotational angle; x is the distance in nm; θ is the RNA pol II rotation angle, and ∅ is the DNA rotation at the RNA pol II site. Based on DNA supercoiling–transcription interplay, experiments show that the maximum RNA pol II translocation rate or velocity is $\upsilon_{0}=20\;\mathrm{nm}.s^{-1}\approx (60\;bp).s^{-1}$ [26].

The ability of the RNA pol II as the scaffold for the PIC arises from its CTD, which is flexible and highly disordered. A close relationship between CTD and phase separation is vital in initiating efficient transcription. CTD-P activates co-transcriptional capping [31]. The favorable regulation of CTD in condensates is reversibly controlled depending on CTD-P [29]. It determines the RNA pol II positioning into different condensates as transcription progresses. For example, the transition between initiation and elongation is associated with Kin_28_-mediated Ser_5_-P within the heptapeptide repeats [27]. Hence, it raises an essential question of whether P-reactions are the transcription’s driving pulse.

In P-reactions, the side chain hydroxyl groups on specific proteins’ serine, threonine, and tyrosine residues are modified with the gamma phosphate, producing a theatrical change in the protein’s architecture and function [62]. ATP is almost always the phosphate donor [63]. It can be represented as A-P~P~P, where °~°°~° are the high-energy acid anhydride bonds. One of those is hydrolyzed. Breaking these bonds is an endergonic process that consumes energy rather than releasing it [64,65]. The negative change in the Gibbs free energy is due to the bonds formed after the hydrolysis process having a lower energy. The new bonds release 7.7 kcal/mol: ΔG = −7.7 kcal/mol o −31 KJ/mol [66].

Unlike most biological processes, in which only one phosphate group ($P_{i}$) is separated, the last two phosphate groups are released in the form of the ${PP}_{i}$ in replication. This process can be summarized in the following chemical equation [67]:

$${\left(DNA\right)}_{n}+dNTP↔{\left(DNA\right)}_{n+1}+{PP}_{i}.$$

Similarly, during transcription elongation, the overall reaction equation [68] is as follows:

$${\left(NMP\right)}_{n}+NTP↔{\left(NMP\right)}_{n+1}+{PP}_{i}.$$

The H-bond is a robust fixed dipole–dipole electrostatic force. It provides excellent stability but is weaker than the covalent or ionic bonds. The energy of a H-bond is typically 5 to 30 kJ/mol, but it can vary from very weak to very strong. For N–H…N, it is approximately 13 kJ/mol. Intermolecular H-bond is responsible for the high boiling point of water (100 °C). ATP to ADP hydrolysis releases 30.5 kJ/mol, with a free energy change of 3.4 kJ/mol [66].

A transition between the TLS energy levels caused by forcing one of the system parameters’ Hamiltonian is called a Landau–Zener transition. A series of those transitions occurs when the periodic forcing has a sufficiently intense amplitude [69]. Detuning can be modified externally:

$$\epsilon \left(t\right)=\epsilon_{0}+Acos\omega_{ext}t$$

where A and $\omega_{ext}$ are the forcing amplitude and frequency, respectively. The qubit levels are anti-symmetrically coupled

$$g_{1}={-g}_{2},\;\mathrm{and}\;\epsilon_{1}\left(t\right)=-\epsilon_{2}\left(t\right).$$

The qubit eigenstates can be defined as |↑⟩, |↓⟩.

The dispersive detuning regime (where $\epsilon \left(t\right)\gg g$) is helpful in quantum computing because it allows for the quantum measurements of the qubit state and its manipulation [11]. Irradiating at the qubit frequency (Ω) handles the qubit coherently. Irradiating at the resonator frequency ($\omega_{r}$) introduces photons in the cavity that become entangled with the qubit states. The law of conservation of linear momentum requires that at least two photons be created so that the resulting linear momentum can be equal to zero [46,70]. The photon is the gauge boson of the electromagnetic interaction and, therefore, is a boson that acts as a carrier of a fundamental interaction of nature. Conservation laws can determine the energies of the two photons or, equivalently, their frequencies. A driving radiation field of frequency $\omega_{ext}$ acting on the qubit–resonator system during a specific time can be described by the Hamiltonian [71]:

(11) $$\hat{H}\left(t\right)=\hbar ∆\left(t\right)\left({\hat{b}}^{+}e^{-i\omega_{ext}t}+{\hat{b}}^{-}e^{i\omega_{ext}t}\right)$$

Every qubit is represented as a point on the Bloch sphere, where the poles represent the eigenstates [72]. The colatitude angle, θ, runs through the angles from 0 to π, while the azimuthal angle, ϕ, runs from 0 to 2π [73]. The effect of a quantum gate on a single qubit is a rotation in the Bloch sphere, giving rise to a new qubit. In the Bloch sphere, we visualize the action of different logic gates or the temporal evolution of the state of a TLS described by a Hamiltonian. An axis and a rotation angle define a rotation operator [74]. Its action produces a rotation of the point on the sphere concerning the rotation axis at the rotation angle [73,75]. The relationship between the angle of rotation in the x-axis and the pulse parameters in the Bloch sphere is represented in the following equation:

$$\theta \left(t\right)=\Omega V_{0}\int_{0}^{t}f\left(t^{\prime }\right)dt^{\prime }$$

Since the Rabi oscillations follow an expression of sin^2^, we can obtain their period and determine the Rabi frequency. Even more importantly, the value of the pulse variable parameter (makes it go from the state |0⟩ to the state |1⟩, a logic gate π) can be evaluated [75]. To perform rotations around the y-axis is enough to multiply the pulse amplitude by the imaginary unit (a phase of $\emptyset =\frac{\pi }{2}$ be added to the pulse).

$$U\left(t\right)=exp\left(-i\int_{0}^{t}H_{d}^{I}\left(t^{´}\right)dt^{´}\right)=exp\left(\frac{i}{2}\Omega V_{0}\left(I\sigma_{x}-Q\sigma_{y}\right)\int_{0}^{t}f\left(t^{´}\right)dt^{´}\right)$$

By combining Equation (11) with the Hamiltonian of the system in Equation (9) and applying the unitary transformation [52]

$$\hat{U}=exp\left(\frac{g}{\epsilon \left(t\right)}\left({\hat{b}}^{-}\sigma_{+}-{\hat{b}}^{+}\sigma_{-}\right)\right),$$

we obtain the single-qubit Hamiltonian in the frame rotating at the driving radiation field of frequency $\omega_{ext}$ acting on the qubit–resonator system during a specific time:

$$\hat{H}=\frac{\hbar }{2}\left(\Omega +2\frac{g^{2}}{\epsilon \left(t\right)}\left({\hat{b}}^{+}{\hat{b}}^{-}+\frac{1}{2}\right)-\omega_{ext}\right)\sigma_{z}+\hbar \frac{g∆}{\epsilon \left(t\right)}\sigma_{x}+\hbar \left(\omega_{r}-\omega_{ext}\right){\hat{b}}^{+}{\hat{b}}^{-}+\hbar ∆\left({\hat{b}}^{+}+{\hat{b}}^{-}\right)$$

The current in a transmission line is proportional to the relationship between the voltage’s magnitude and the line’s impedance (I = V/Z). This simple Ohm’s Law will hold for a transient period [76].

Three effects can generate a superconducting phase difference around the circuit [11]:

1. Due to the Josephson effect, when the pairs coherently pass through a JJ, the supercurrent Ι related to the phase difference between electrodes can be represented by $Ι={Ι}_{0}sin\phi$. ${Ι}_{0}$ is the maximum critical current the junction can maintain. In our model of DNA, the maximum crucial current of the H-bond is ${Ι}_{0}$;
2. Due to the supercurrent, an intrinsic phase shift appears when computing the line integral of momentum $\vec{p}$ pairs moving along the curve;
3. Due to external magnetic flux, classical electromagnetism and Maxwell’s laws show that a magnetic field that changes with time induces an electric field. This, in turn, modifies the momentum of the charged particles by exerting a force on them. The moment of the electron pairs is given by equation $\vec{p}=2m_{e}\vec{v}+2e\vec{A}$. The first component corresponds to the kinetic part, and the second to the field contribution. The potential vector is $\vec{A}$.

RNA pol II changes the external magnetic flux through a P-mechanism. It generates a phase difference by sending a pair of pulses with the same parameters many times (n). The total number of pulses required may explain why the enzyme’s catalytic site has 52 repeated heptad motifs (Tyr_1_Ser_2_Pro_3_Thr_4_Ser_5_Pro_6_Ser_7_) (Figure 1A). Then, the number of times (m) the system was in the state |0⟩ or |1⟩ is extracted. The probability that the system is in a specific state is calculated by applying Laplace’s rule. For example, to obtain the probability that it is in state |0⟩: $P\left(\left|0\right\rangle \right)=\frac{m}{n}$. We can obtain the Rabi oscillations by representing the probabilities as a function of time. At a computational level, a pulse is a finite time series of complex values, where each value represents a pulse amplitude at a given time. In DNA, a pulse is a finite time series of P-reactions.

## 3. Discussion

The spin 1/2 particles are TLS, useful for quantum computation [77]. Previously, close connections between classical and quantum information were described in DNA [8]. In this work, we characterized A–T and C–G bps as flow qubits, as they constitute a superconducting ring interrupted by one JJ and crossed by a magnetic flux. Recent studies have treated the H-bonds between DNA bps as adiabatic systems with spin–orbit coupling. Hubač and Cols extensively defined qubits formed by Majorana fermions in the H-bond and discussed the entangled states in the bps [78]. The Hamiltonian obtained in this work for the DNA qubits differs from that of the transmon. However, in the limit when L tends to be infinite (omitting the term $\frac{1}{2L}$ in Equation (2)), our Hamiltonian (Figure 2B) tends to be the transmon Hamiltonian (Figure 2A).

In DNA, adjacent bases are connected by the sugar–phosphate backbone. This structure is similar to a transmission line with distributed parameters. Changes in the magnetic field by the RNA pol II during transcription could flip nuclear spins at resonance. An applied magnetic field breaks electrons’ twofold spin degeneracy. A pulse set detuned from the resonant frequency enables the state of the qubit to be determined. One question emerged, “What is the energy required to achieve the lowest donor-excited state?” This work considered high intra-complex P-activity manifested in processive P-reactions of alcohol residues in RNA pol II, using ATP as the phosphate donor as the RNA pol II driving force. Multisite P-networks emerge as a new level of signal based on routes encoded into disordered regions of proteins [79]. PPi release during transcription is a signature step in each nt addition cycle [68]. A DNA circuit can be represented as a symmetric and antisymmetric sin function: y=Asinx  and y=−Asinx. We proposed the graphic representation of the DNA strands’ deformation due to the H-bond break to form the Bloch sphere. The rotations of the qubit are carried out during transcription (Figure 1B).

The coupling of each spin to its neighbors and the magnetic field enables numerous operations on each spin. Measurements are performed by determining the electron spin state effect on the electrons’ orbital wavefunction after transferring nuclear spin polarization to the electrons [11]. Also, quantum computation requires a two-qubit controlled rotation proceeding. The result is a rotation of a target qubit when the control qubit is oriented in a specified direction and still unchanged [20]. The measurable signal decreases with the number of qubits [20,54,80]. Previous studies reported that more than ten qubits will be challenging [80]. Looking at the spins inside a DNA loop, they are arranged in parallel. In principle, logical operations and measurements can be performed independently and in parallel on each spin in the array.

Circuit QED points to multiple superconducting qubits and 3D cavities with high coherence [21]. Distributed quantum computation arises across numerous single-mode resonators [20]. Single-step multi-qubit phase gates on multiple single-mode resonators mediated by a superconducting bus in circuit QED have been described [81]. The superconducting bus may be possible single-mode resonator interactions, and quantum information is encoded in various single-mode resonators’ vacuum and single-photon states [11]. In addition, the pulse engineering technique adjusts the coupling strength between resonators and the bus, enhancing fidelity and robustness. The tunable coupling strength plays a broad role in the circuit-QED system [20].

Based on the above information, we consider that a DNA gene’s basic structure is the nonperturbative circuit QED, and a complete gene would be the linear combination of n circuits in parallel. This parallel arrangement, together with its superconducting nature, contributes to the total resistance being zero, and there is no heat dissipation. DNA gene transcription process includes the combination of n cavities hosting nine qubits and coupled to a shared machine. Many qubits in a single cavity may increase the unwanted qubit–qubit interaction and the cavity decay while decreasing the qubit–cavity coupling strength [20,54,80]. Hence, multiple cavities and quantum operations on qubits distributed in different cavities are mandatory for large-scale quantum information processing.

The RNA pol II bus holds a level structure formed by ground and excited levels, denoted by $\left|g_{1},\;g_{2}\ldots g_{n}\right\rangle$ and $\left|e_{1},\;e_{2}\ldots e_{n}\right\rangle$, respectively. Adjusting the qubit level spacings achieves the coupling and decoupling of each qubit from its cavity. The P-pulses applied to the bus drive the transitions resonantly between $\left|e_{x}\right\rangle$ and $\left|g_{x+1}\right\rangle$, with Rabi frequency $\Omega_{x}$. Realizing quantum information transfer (QIT) and entanglement with SQUIDs in a microwave cavity is feasible in artificial atom models [82].

## 4. Conclusions

Biomolecular condensates concentrate functionally related components through multivalent and dynamic interactions in biopolymers without a bounding membrane. Using the transcription condensate architecture and the Electrodynamic Quantum Theory of Circuits, we further developed ideas about the electrical design of quantum information in DNA. This theory is an excellent model for characterizing the transcription process and describing the Hamiltonians that define the function in secondary quantification using the methods of Quantum Physics. In this work, we address the graphic and physical–mathematical representation of the DNA qubit as a nonlinear quantum circuit that introduces an anharmonicity through the H-bond. There are similarities between DNA qubits and RF SQUIDs coupled to a cavity mode defined by two resonators. However, DNA qubits differ from the transmon qubits.

To characterize DNA as a transmission line or a wire model, the backbone structure of single-stranded DNA must be analyzed. It has connections between successive bps. Hence, the line’s superconductors in a given gene are the bps. Each single DNA strand backbone works like a TEM with discrete distributed parameters. In this work, we described the Hamiltonian of one qubit coupled to two resonators. We concluded that every gene in DNA can be modeled as a combination of (n) circuits. The RNA pol II, acting like a multifunctional biomolecular machine, produces the system’s decoherence like an artificially engineered device. A processive P circuit with multiple kinase inputs and in the 52 repeated heptad motif (Tyr_1_Ser_2_Pro_3_Thr_4_Ser_5_Pro_6_Ser_7_) controls multi-target qubits. The PPi release step is crucial to the mechano–chemical coupling mechanism during transcription elongation because it is equivalent to the computation pulse. The transcription process involves coupling (n) circuits, each containing nine qubits.

Evolutionarily conserved biological processes are critical and contain essential secrets that allow for a specific function to be carried out. Knowing these deep secrets helps us understand fundamental human and natural development processes. Understanding basic quantum interactions is essential to discovering these biological secrets. Quantum Molecular Biology is an emerging and fascinating field of study. The quantum information in DNA is related to life evolution and preservation.

## Data Availability

Data are contained within the article.
